# Effect of Pre-Harvest Sprouting on Physicochemical Properties of Starch in Wheat

**DOI:** 10.3390/foods3020194

**Published:** 2014-04-08

**Authors:** Senay Simsek, Jae-Bom Ohm, Haiyan Lu, Mory Rugg, William Berzonsky, Mohammed S. Alamri, Mohamed Mergoum

**Affiliations:** 1 Department of Plant Sciences, North Dakota State University, P.O. Box 6050, Department #7670, Fargo, ND 58108-6050, USA; E-Mails: haiyan.lu22@outlook.com (H.L.); mory.O.P.Rugg@gmail.com (M.R.), mohamed.mergoum@ndsu.edu (M.M.); 2 USDA-ARS Hard Red Spring and Durum Wheat Quality Laboratory, Harris Hall, North Dakota State University, Fargo, ND 58108, USA; E-Mail: jae.ohm@ars.usda.gov; 3 Department of Plant Sciences, South Dakota State University, Brookings, SD 57007-2141, USA; E-Mail: bill.berzonsky@bayer.com; 4 Nutrition and Food Sciences Department, College of Food and Agricultural Sciences; King Saud University, P.O. Box 2460, Riyadh 11451, Saudi Arabia; E-Mail: msalamri@ksu.edu.sa

**Keywords:** pre-harvest sprout, wheat, starch, HPSEC

## Abstract

Pre-harvest sprouting (PHS) in wheat (*Triticum aestivum* L.) occurs when physiologically mature kernels begin germinating in the spike. The objective of this study was to provide fundamental information on physicochemical changes of starch due to PHS in Hard Red Spring (HRS) and Hard White Spring (HWS) wheat. The mean values of α-amylase activity of non-sprouted and sprouted wheat samples were 0.12 CU/g and 2.00 CU/g, respectively. Sprouted samples exhibited very low peak and final viscosities compared to non-sprouted wheat samples. Scanning electron microscopy (SEM) images showed that starch granules in sprouted samples were partially hydrolyzed. Based on High Performance Size Exclusion Chromatography (HPSEC) profiles, the starch from sprouted samples had relatively lower molecular weight than that of non-sprouted samples. Overall, high α-amylase activity caused changes to the physicochemical properties of the PHS damaged wheat.

## 1. Introduction

Pre-harvest sprouting (PHS) occurs as a result of the germination of kernels within the wheat spike before harvest, and it can often occur when harvest coincides with relatively high humidity due to untimely rain [1]. PHS generally occurs after maturation of the wheat kernel, however germination may occur as early as 18 days after anthesis [2]. Symptoms of PHS include premature kernel swelling, germ discoloration, seed-coat splitting, and root and shoot emergence [1]. The severity of PHS can also be classified on a continuum from very minor to very severe, which differs from year-to-year, depending on the weather [3,4]. PHS can be measured by calculating the percentage of sprouted wheat kernels and by measuring starch degradation, which is commonly quantified by the falling number test as an indirect measurement of α-amylase activity in grain [4]. PHS may result in monetary losses to growers, millers and bakers [3]. The price of sprouted wheat could be reduced by 20%–50%, and the grain is classified as unacceptable for human food if it contains more than 4% damaged (sprouted) kernels [5]. Severely sprouted grain is often used only as animal feed. Millers experience losses because of an associated reduction in flour yield and quality as well as functional quality. Bakers can experience problems during the bread-making process and suffer losses because the end-product is of poor quality. Products made from flour containing sprouted wheat are porous, sticky, and off-color and generally of poor bake quality [5].

In this study, we investigated physicochemical properties of starch from non-sprouted and sprouted hard red spring (HRS) and hard white spring (HWS) wheat samples. Though, there are many complex factors, such as amylases, amylase genes, abscisic acid (ABA), ABA genes and other genes (VP1) [6,7], dormancy and susceptibility to PHS are also related to seed coat color. Most of the HRS wheat genotypes released from breeding programs display a high level of tolerance to PHS [4,8]. Although many studies have shown that hard white wheat genotypes are more susceptible to PHS, there is considerable variability in the level of tolerance to PHS in wheat [4,8]. Pleiotropic effect of *R* (*Red grain colour*) genes conferring red pericarp color and other dormancy genes (has a major effect on the embryo) affect the wheat grain dormancy [8,9]. Thus, PHS in wheat is controlled by coat-imposed and embryonic pathways regulated by separate genetic systems [6,9,10]. Red wheat tends to have higher levels of PHS resistance than white wheat; although, some studies have shown that sources of resistance to PHS can be acquired in white wheat germ plasm [4,8,11].

A new plant requires energy and nutrients, which is the reason why a sprouting wheat kernel produces amylases, lipases and proteases. These enzymes break down starch, oil and protein to feed the developing embryo [12,13]. The impact of PHS on the final food product depends on the amount of enzymes present and their break down of kernel starches, oils, and proteins [5]. Partial hydrolysis of starch leads to a decrease in the size of the starch molecules and a reduction in the water-holding capacity of the dough. Few studies have documented the physicochemical changes to wheat starch due to PHS. However, the altered pasting, gelation, and retrogradation properties of starch [14], can significantly affect the quality of products such as Asian noodles, sponge cake, and breads.

Starch is the main storage polysaccharide and energy source for plants. Wheat flour is composed of 70%–80% dry matter of starch. Compared to other carbohydrates, starch possesses unique physical and chemical properties [15]. In wheat, there are two main types of starch granules: large, lenticular (A type) and small, spherical (B type). Starch granules are comprised of two constituent polymers: amylose a basically linear polysaccharide (α-1→4 linked glucose), and a highly branched polysaccharide termed amylopectin (α-1→4 linked glucose and α-1→6 linked glucose) [15]. Amylopectin is the major component of starch and normal wheat starch is comprised of about 75% amylopectin. The distribution of starch granule types and structure of amylose and amylopectin and their relative ratios in starch granules play an important part in determining pasting, gelation, and retrogradation properties of starch and end-product quality and stability [16].

## 2. Materials and Methods

### 2.1. Materials

Wheat samples were kindly provided by the Spring Wheat Breeding Programs in the Plant Sciences Department at North Dakota State University 24 genotypes, including 12 hard red spring (HRS) and 12 hard white spring (HWS) wheat genotypes grown at three locations (Table 1). “Hanna” and “AC Snowbird” have previously demonstrated a high level of seed dormancy [4]. Therefore, Hanna was considered a HRS and AC Snowbird a HWS control for tolerance to PHS. “Ingot” and “Lolo”, previously exhibiting susceptibility to PHS, were considered PHS susceptible HRS wheat and HWS wheat types, respectively. The lines selected as check samples were chosen because they are well characterized and their response to PHS has been determined and previously documented in other research [4].

Experimental plots were grown at Prosper, Carrington, and Casselton, ND in 2008. In each location, the experiment was laid out in a randomized block design with four replicates following the procedure of Rugg [4]. Environmental conditions with respect to temperature and rain in the 2008 growing season were as follows: Each location received lower than average precipitation through the entire growing season. Temperatures were below normal at both environments in the early part of the growing season. Prosper experienced higher precipitation and higher temperatures compared to Carrington, which resulted in higher disease pressure in Prosper than in Carrington [4].

In this study, sprouted and non-sprouted wheat samples were analyzed in two replicates. Two samples from 4 field replicates were combined to produce the 2 replicates used for analysis. Thus, physicochemical characterization of starch from a total of 288 samples (24 entries × 2 replicates × 3 locations × 2—sprouted and non-sprouted) was analyzed in the present research.

### 2.2. Sample Sprouting and Sprout Score

Wheat samples were evaluated and scored for tolerance to PHS by the Department of Plant Science, North Dakota State University. At plant physiological maturity, 30 wheat spikes were randomly harvested from each experiment unit. There was no PHS damage evident at the time of harvest. To inhibit additional α-amylase activity the spikes were immediately stored at 10 °C. The spikes were placed in a mist chamber and misted for a period of 48 h. Following the misting, a humidifier was placed in the chamber for 3 days. Visual observations of the spikes were made to assess the degree of sprouting induced by maintaining high moisture in the misting chamber. Spikes were scored visually 0–9; whereby 0 represented no visible sprouting and a score of 9 represented very severe sprouting with average coleoptiles lengths greater than 2 cm [4].

**Table 1:** Sprouting score and α-amylase activity of sprouted wheat, non-sprouted wheat and their difference (ΔD).

Genotype	Sprout Score	α-Amylase Activity (CU/g)		
		Sprouted	Non-Sprouted	ΔD
RSW	H	-	-	-
Hanna	2.8	1.32	0.11	1.20
Ingot	7.0	2.37	0.10	2.27
Alsen	4.8	1.82	0.09	1.57
Briggs	5.7	2.16	0.11	2.06
Freyr	4.4	1.79	0.09	1.70
Glenn	4.0	1.68	0.08	1.61
Granite	5.3	2.09	0.13	1.96
Kelby	3.4	1.56	0.13	1.43
Norpro	6.0	2.18	0.09	2.09
Reeder	4.4	1.76	0.08	1.68
Steele-ND	5.0	1.93	0.08	1.85
Knudson	5.4	2.12	0.10	2.02
Mean	4.8	1.90	0.10	1.79
*HWSW*				
99S0155-14W	2.5	1.36	0.12	1.24
Otis	7.8	2.47	0.11	2.37
AC Snowbird	2.8	1.39	0.08	1.31
AC Vista	5.8	2.13	0.09	2.04
Argent	4.8	1.98	0.16	1.83
CS3100L	6.8	2.33	0.18	2.16
CS3100Q	6.8	2.44	0.14	2.3
Explorer	6.9	2.37	0.16	2.21
Lolo	5.7	2.17	0.12	2.05
MT9420	6.9	2.33	0.14	2.19
NDSW0602	6.3	2.37	0.12	2.24
Pristine	5.0	1.99	0.18	1.81
Mean	5.7	2.11	0.13	1.98
LSD	1.4	0.39	0.05	0.40

ΔD: Difference between non-sprouted and sprouted wheat; LSD: least significant difference (α = 0.05), used to detect difference between genotypes.

### 2.3. α-Amylase Activity

Wheat samples were dried and ground in a cyclone sample mill (Udy, Fort Collins, CO, USA) equipped with a 1 mm sieve. Samples of ground wheat (0.5 g) were weighed into test tubes containing stir bars. The test tubes were placed into a stirring heat block at 60 °C and stirred at medium high speed. Sodium maleate buffer (5 mL, 100 mM, pH 6.0) was heated to 60 °C and added to each tube, stirred for 5 min and then an amylazyme tablet (Megazyme, International, Ireland) was added. The reaction was stopped by adding 6 mL Trizma base (2% w/v, pH 9.5) after 5 min. Subsequently, the sample was left at room temperature for 5 min, then stirred and filtered. The absorbance of the filtrate at 590 nm was measured against the reaction blank and α-amylase activity was calculated by reference to a standard curve.

### 2.4. Pasting Properties

Pasting properties of samples were evaluated by using a RVA (Newport Scientific, Narrabeen, Australia) according to AACC approved method 76-21.01 [17]. Samples of ground wheat (3.5 g, 14% moisture basis) were added to pre-weighed, de-ionized distilled water samples in a RVA canister. Parameters of Peak Viscosity (PV), breakdown (BD), Hot Paste Viscosity (HPV), setback (SB) and Final Viscosity (FV) were recorded.

### 2.5. Scanning Electron Microscopy (SEM) Analysis of Starch Granules

Two PHS samples with the highest sprout scores were chosen from both HRS and HWS genotypes. The other two were from non-sprouted samples. Wheat kernels were cracked open longitudinally through the crease and fixed to microscope stubs with Dotite silver paint. The samples were coated with gold using a Hummer II sputter coater (Technics/Anatech Ltd., Alexandria, VA, USA) [18]. Images were obtained using a JEOL JSM-6490LV Scanning Electron Microscope (SEM) (JEOL, Peabody, MA, USA).

### 2.6. High Performance Size Exclusion Chromatography (HP-SEC) Analysis of Starch

Eight samples of ground wheat were chosen to conduct the HPSEC analysis. Among the 8 samples, 4 samples with each of the HWS and HRS (samples were chosen to give a representation of HWS and HRS with high and low sprout scores) were included. Starch samples were analyzed as described by Simsek *et al.* [19].

### 2.7. Statistical Analysis

Statistical analyses were performed using the SAS System for Windows (V. 9.2, SAS Institute, Cary, NC, USA). Bartlett’s test was used to analyze the homogeneity of error variance across the three locations. When error variances were homogenous, analysis of variance (ANOVA) was performed using the “Mixed” procedure in SAS, assuming location as a random effect and genotype as a fixed effect. The difference between the HRS and HWS mean value was analyzed using the “Contrast” option. Bartlett’s test indicated that error variance of pasting properties for sprouted samples were heterogeneous across the three locations. Thus, each location was analyzed separately for pasting properties. Correlation coefficients were calculated across genotype means using the “Corr” procedure in SAS.

## 3. Results and Discussion

### 3.1. α-Amylase Activity in Non-Sprouted and Sprouted HRS and HWS Wheat Samples

In non-sprouted kernels, α-amylase activity is mostly localized to the seed coat, aleurone layer, and scutellum [20]. Based on Bartlett’s test, variances across the samples from the three locations were homogeneous for the properties being measured. Thus, the three locations were combined for analyses.

Alpha-Amylase activity of the wheat samples are given in Table 1. The mean value of α-amylase for sprouted samples was 2.00 CU/g; while this value for non-sprouted was 0.12 CU/g, and all of the sprouted samples had higher α-amylase than non-sprouted. These results were in agreement with the findings of Ichinose *et al.* [13] who reported that the α-amylase activity of wheat increases rapidly as germination progresses. A previous report also indicated that the starch in sprouted wheat samples degraded rapidly as the α-amylase activity increased during PHS [13].

Genotypes tested within sprouted samples had sprout scores ranging from 2.5 to 7.8 (Table 1). In this study, the sprout tolerant and susceptible HRS check samples corresponded to the samples with sprout scores and α-amylase activities at the extremes of the evaluation range. Among sprouted samples, Hanna had the lowest sprout score (2.8), while Ingot had the highest sprout score (7.0). The α-amylase activities of sprouted samples of Hanna and Ingot were 1.32 CU/g and 2.37 CU/g, respectively. The check samples for the sprout tolerant (AC Snowbird) and susceptible (Lolo) HWS genotypes were at the low and high end of the sprout score and α-amylase activity range, respectively, but they did not exhibit the highest or lowest values. Breeding line 99S0155-14W had the lowest sprout score at 2.5, while Otis had the highest sprout score (7.8) among sprout damaged HWS samples. Among sprouted samples, the α-amylase activity of 99S0155-14W and Otis were 1.36 CU/g and 2.47 CU/g, respectively. These results agreed with the findings of Huang [21], who reported that genotypes with lower susceptibility to PHS also had lower α-amylase activity.

Wheat genotypes have high α-amylase activity without visible sprouting. The relationship between sprout score and α-amylase activity of each genotype was determined (Table 1). There was a significant difference in α-amylase activity and ΔD among genotypes within sprouted samples (α = 0.05). This indicated that varietal differences were highly significant for α-amylase activity. α-amylase activity may be a better indicator—than sprout score—as to which genotypes have low tolerance to PHS. The differences in α-amylase activity between the three locations were not as great as those between genotypes. Interactions between genotypes and location were significant, suggesting that environments affect PHS and enzyme activity, but not as much as the genotype.

### 3.2. Pasting Properties of Non-sprouted and Sprouted HRS and HWS Wheat

The pasting parameters for the check samples and the averages of all varieties in each wheat class for each location of sprouted and non-sprouted wheat samples and their ΔD are shown in Table 2. All RVA properties changed dramatically due to PHS, which suggests PHS also significantly impacted starch pasting properties. All sprouted samples had much lower peak viscosity, hot paste viscosity, and final viscosity than non-sprouted samples. Peak viscosity of non-sprouted samples ranged from 138.9 to 289 RVU (data not shown). Peak viscosity of sprouted wheat ranged from 5 to 18 RVU. These results suggest that the water binding capacity of starch and starch paste stability decreased due to PHS. The pasting profile of starch determined by RVA has a direct relationship to the microstructure of the starch. Amylose, which is important to high gel consistency upon cooling, may contribute to determining the initial rigidity of swollen starch granules during germination [22]. The branch chain length of amylopectin has an effect on the gelatinization, retrogradation, and pasting properties of starch [16].

Starch granules can lose their resistance to swelling due to higher activity of α-amylase [12], and the reduced resistance to swelling may in turn have lowered the paste viscosity of the sprouted samples. Genotypes with higher tolerance to PHS had higher water binding capacity and higher starch paste stability.

Table 3 shows the correlations per location between pasting characteristics, sprout score, and α-amylase activity among the 24 genotypes. The correlations between sprout score and peak viscosity were negative and highly significant for sprouted samples from Casselton and negative and very highly significant for sprouted samples from Carrington and Prosper. The correlations for sprouted samples between sprout score and peak viscosity were −0.64, −0.56 and −0.76 for Carrington, Casselton and Prosper, respectively. Furthermore, correlations between sprout score and the ΔD of hot paste viscosity was significant for samples grown in Carrington (−0.48) and Casselton (−0.43), which is a consequence of genotypes with high sprout scores also exhibiting low peak viscosity. Significant and negative correlations were obtained between the α-amylase of non-sprouted samples from Carrington. For non-sprouted samples from Casselton and Prosper, correlations between α-amylase activity and peak viscosity and α-amylase and hot paste viscosity were negative and very highly significant. The correlation between α-amylase activity and final viscosity for non-sprouted samples from Casselton and Prosper was negative and highly significant. There were significant negative correlations between α-amylase activity of sprouted samples and peak viscosity of sprouted samples from Carrington (−0.61), Casselton (−0.54) and Prosper (−0.69). Thus, the pasting viscosity of sprouted wheat had also been decreased as a result of PHS.

**Table 2:** Mean value of pasting profile of check varieties; Hanna, Ingot, Lolo and AC Snowbird; and means of wheat classes from Carrington, Casselton and Prosper for sprouted wheat, non-sprouted wheat and their difference (ΔD).

Wheat Class	Genotype	Peak Viscosity			Hot Paste Viscosity			Final Viscosity		
		Sprouted	Non-Sprouted	ΔD	Sprouted	Non-Sprouted	ΔD	Sprouted	Non-Sprouted	ΔD
*Carrington*										
**HRSW**	Hanna	11.2	222.7	212.8	2.3	134.8	133.2	3.5	258.5	255.4
	Ingot	3.5	233.4	230.4	2.3	139.9	137.6	2.9	256.7	253.8
	Mean	4.4	224.4	221.4	1.4	126.5	125.6	1.7	244.5	243.1
**HWSW**	AC Snowbird	11.4	252.4	239.3	1.9	125.2	124.2	3.0	237.8	235.5
	Lolo	2.2	210.4	205.4	0.6	93.0	90.8	0.6	202.9	200.3
	Mean	3.1	209.3	207.1	2.0	99.2	97.8	2.4	215.7	213.9
	**LSD**	1.8	19.3	19.5	1.3	18.1	18.6	1.3	28.2	28.6
*Casselton*										
**HRSW**	Hanna	20.5	197.9	177.4	3.1	128.6	125.4	4.8	237.5	232.4
	Ingot	10.6	210.1	198.6	2.5	133.6	130.6	3.1	233.2	229.6
	Mean	12.4	204.5	192.3	2.3	122.0	119.5	3.1	223.1	219.8
**HWSW**	AC Snowbird	14.8	242.0	228.6	1.9	129.8	128.3	3.1	236.1	233.8
	Lolo	10.6	213.9	200.0	2.8	112.0	108.4	3.5	216.2	212.0
	Mean	10.6	188.9	177.5	2.6	94.0	91.3	3.3	193.7	190.1
	**LSD**	5.3	25.5	25.4	2.1	20.4	19.9	2.8	37.9	37.4
*Prosper*										
**HRSW**	Hanna	14.7	200.2	183.2	1.8	129.1	125.6	3.0	240.8	236.2
	Ingot	6.8	236.2	227.6	2.0	153.7	150.4	2.6	274.4	270.6
	Mean	8.4	201.7	192.9	2.0	119.9	117.8	2.4	228.2	225.9
**HWSW**	AC Snowbird	17.9	289.0	268.2	1.9	163.7	160.9	3.3	294.6	290.5
	Lolo	8.6	203.5	193.8	3.5	103.5	100.8	3.7	209.6	206.7
	Mean	8.1	189.8	180.8	2.3	95.1	92.4	2.8	203.3	200.3
	**LSD**	2.3	24.1	24.3	1.2	19.5	19.3	1.3	30.9	30.8

Only the two check varieties are shown with the means for each wheat type at each location and the LSD for each location; The unit is expressed by Rapid Viscosity Unit (RVU); ΔD: Difference between non-sprouted and sprouted wheat; LSD: least significant difference (α = 0.05).

**Table 3:** Correlation coefficients ^a^ between α-amylase activity and pasting characteristics among 24 genotypes from Carrington, Casselton and Prosper.

Pasting Characteristics		Sprout	Sound	PHS	∆D
		Score	α-Amylase	α-Amylase	α-Amylase
*Carrington*					
Non-Sprouted	Peak Viscosity	NS	−0.45 *	NS	NS
	Hot Paste Viscosity	−0.47 *	−0.50 *	−0.51 *	−0.45 *
	Final Viscosity	NS	NS	−0.43 *	NS
Sprouted	Peak Viscosity	−0.64 ***	NS	−0.61 **	−0.60 **
∆D	Peak Viscosity	NS	−0.45 *	NS	NS
	Hot Paste Viscosity	−0.48 *	−0.50 *	−0.51 *	−0.45 *
	Final Viscosity	NS	NS	−0.43 *	NS
*Casselton*					
Non-Sprouted	Peak Viscosity	−0.41 *	−0.67 ***	NS	NS
	Hot Paste Viscosity	−0.44 *	−0.68 ***	NS	NS
	Final Viscosity	NS	−0.54 **	NS	NS
Sprouted	Peak Viscosity	−0.56 **	NS	−0.54 **	−0.54 **
∆D	Peak Viscosity	NS	−0.68 ***	NS	NS
	Hot Paste Viscosity	−0.43 *	−0.69 ***	NS	NS
	Final Viscosity	NS	−0.55 **	NS	NS
*Prosper*					
Non-Sprouted	Peak Viscosity	NS	−0.64 ***	NS	NS
	Hot Paste Viscosity	NS	−0.68 ***	NS	NS
	Final Viscosity	NS	−0.57 **	NS	NS
Sprouted	Peak Viscosity	−0.76 ***	NS	−0.69 ***	−0.68 ***
∆D	Peak Viscosity	NS	−0.66 ***	NS	NS
	Hot Paste Viscosity	NS	−0.69 ***	NS	NS
	Final Viscosity	NS	−0.57 **	NS	NS

^a^ Correlation coefficient is significant at * *p* < 0.05, ** *p* < 0.01 and *** *p* < 0.001, respectively; NS, not significant; ΔD: difference between non-sprouted and sprouted wheat; variables with no significant correlation to any other variable were omitted from the table.

### 3.3. Starch Granule Morphology of Non-sprouted and Sprouted HRS and HWS Wheat

**Figure 1:**
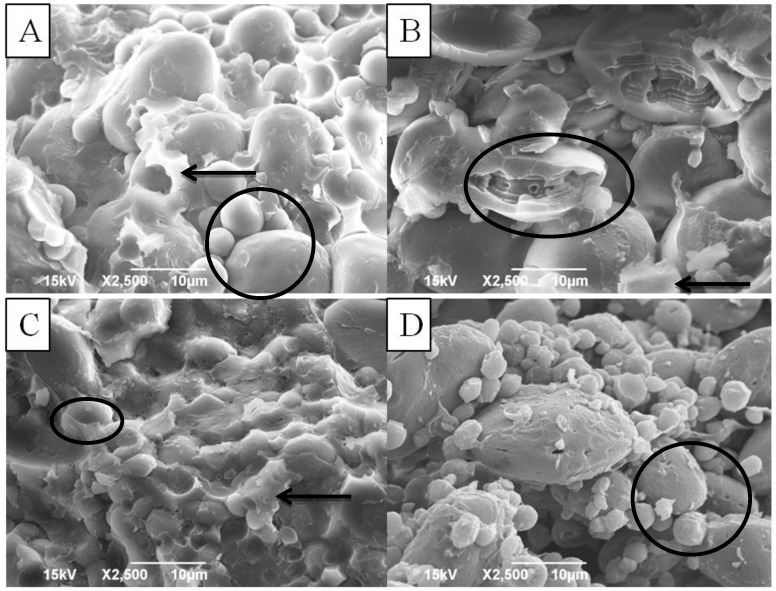
Scanning Electron Microscopy (SEM) images of starch from genotypes Steel-ND and Pristine. (**A**) Image from hard red spring (HRS) wheat genotype Steel-ND non-sprouted sample; (**B**) image from HRS wheat genotype Steel-ND sprouted sample; (**C**) image from hard white spring (HWS) wheat genotype Pristine non-sprouted sample; and (**D**) image from HWS wheat genotype Pristine sprouted sample. Starch granules are circled and protein matrix is identified by arrows.

Starch is deposited in the endosperm of the wheat kernel and is comprised of discrete granules [23]. The endosperm of wheat kernels contains two types of granules—a larger type, mostly about 20–35 mm in diameter (A-starch) and lenticular in shape; and a smaller spherical type, ranging from 2 to 8 mm in diameter (B-starch) [23]. Scanning electron microscopy (SEM) can be used to determine the distribution of the A and B type granules as well as their degradation due to high α-amylase activity. In the endosperm of non-sprouted wheat kernels, starch granules are usually embedded in a dense protein matrix. Artificial sprouting of barley and corn for seven days demonstrated that extensive damage to starch granules can take place due to an increase in α-amylase activity [24]. Dronzec *et al.* [25] and Bean *et al.* [14] have shown extensive damage to wheat starch granules due to PHS. Two genotypes, Steele-ND and Pristine, were chosen to conduct SEM analyses because of their high sprout scores (Table 1) under conditions of PHS. The SEM images of the sprouted and non-sprouted samples from the two genotypes are shown in Figure 1. The non-sprouted samples (Figure 1A,C) exhibited intact starch granules embedded in a very dense protein matrix. Conversely, starch granules had been degraded, and the protein matrix was absent in the sprouted samples (Figure 1B,D). There was also pitting observed on the starch granules of the sprouted samples, most likely due to an increase in α-amylase activity. Similarly, Huang *et al.* [21] reported that the protein matrix was missing or compromised in sprouted wheat samples. The same study demonstrated that proteolytic enzymes broke down the protein matrix, thereby producing a loose structure around the starch granules, which made them more accessible to α-amylase.

### 3.4. HPSEC of Starch in Non-sprouted and Sprouted HRS and HWS Wheat

**Figure 2:**
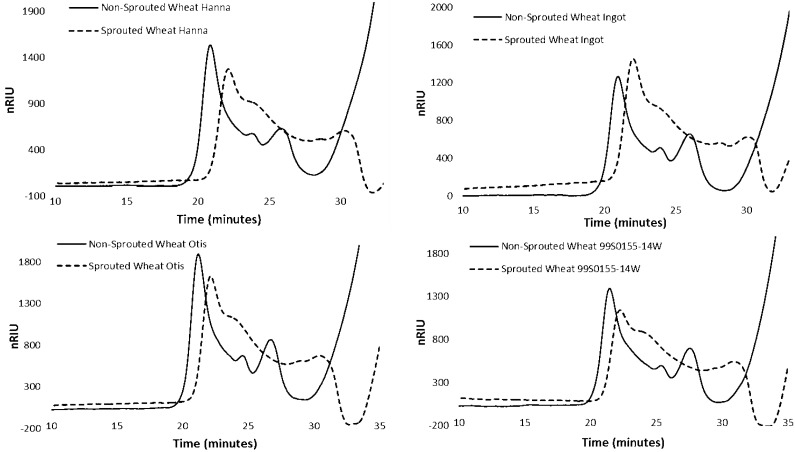
High Performance Size Exclusion Chromatograph (HPSEC) profiles of sound and PHS damaged wheat samples of genotypes Hanna and Ingot (hard red spring wheat genotypes), Otis and 99S0155-14W (hard white spring wheat genotypes). Dashed line represents the HPSEC chromatography of pre-harvest sprouting (PHS) damaged sample; solid line represents the HPSEC chromatography of sound sample.

There were three peaks detected in the HPSEC chromatogram of starch in sprouted and non-sprouted samples (Figure 2), a high molecular weight amylopectin (HMW-AP) peak, a low molecular weight amylopectin (LMW-AP) peak, and an amylose (AM) peak [19]. A comparison of the HPSEC chromatography profiles of sprouted and non-sprouted samples of Hanna, Ingot, 99S0155-14W and Otis, showed that sprouted samples had lower HMW-AP than non-sprouted wheat samples. There was a shift from HMW-AP to LMW-AP and AM, and an overall shift from amylopectin to AM starch in the endosperm of sprouted samples. Thus, the apparent AM content seemed to increase in sprouted samples (Figure 2).

The percentages of each starch fraction and apparent average molecular weights determined by HPSEC are given in Table 4. The large starch molecules were degraded to smaller molecules due to the higher α-amylase activity in sprouted samples. Furthermore, more starch degradation occurred in genotypes that received higher PHS scores. Among sprouted samples, there was a significant decrease in HMW-AP for Hanna (61.5%–31.6%), Ingot (60.6%–34%), Otis (62.8%–32.9%) and 99S0155-14W (64.5%–28.9%), and this hydrolysis of amylopectin resulted in an apparent increase in the percentages of LMW-AP and amylose in the sprouted samples (Table 4). Based on these changes, we presume that starch had been hydrolyzed during PHS due to an increase in α-amylase activity, causing some portion of HMW-AP starch to be converted into LMW-AP and amylose. The structure of amylose and amylopectin and their relative ratios in starch granules play an important part in determining pasting, gelation, and retrogradation properties of starch and end-product quality and stability [16]. Degradation of the amylopectin changes the functional properties of the starch by altering the molecular weight and chain length distribution. The overall decrease in apparent average molecular mass of the starch and the increase of LMW-AP and amylose contents likely caused the reduction in paste viscosity [14] seen in Table 2.

**Table 4:** Percent of starch fractions and molecular weight distribution (MWD) of HMW-AP, LMW-AP and AM from genotypes Hanna and Ingot (HRSW), Otis and 99S0155-14W (HWSW).

Treatment	Genotype	HMW-AP		LMW-AP		AM	
		(%)	MWT	(%)	MWT	(%)	MWT
Non-Sprouted	Hanna	61.5	1.68 × 10^7^	11.8	4.61 × 10^6^	26.8	1.82 × 10^6^
	Ingot	60.6	1.57 × 10^7^	12.8	4.08 × 10^6^	26.6	1.60 × 10^6^
	Otis	62.8	1.41 × 10^7^	11.1	2.99 × 10^6^	26.1	1.15 × 10^6^
	99S0155-14W	64.5	1.26 × 10^7^	11.0	2.10 × 10^6^	24.6	0.80 × 10^6^
	**LSD**	1.2	1.03 × 10^6^	1.1	6.25 × 10^5^	0.1	1.71 × 10^5^
Sprouted	Hanna	31.6	0.95 × 10^7^	40.8	5.21 × 10^6^	27.5	0.24 × 10^6^
	Ingot	34.0	1.01 × 10^7^	38.2	5.46 × 10^6^	27.8	0.27×10^6^
	Otis	32.9	0.95 × 10^7^	37.5	4.90 × 10^6^	29.7	0.24 × 10^6^
	99S0155-14W	28.9	0.92 × 10^7^	42.2	4.88 × 10^6^	28.9	0.19 × 10^6^
	**LSD**	0.8	7.60 × 10^4^	1.7	1.89 × 10^5^	1.1	7.71 × 10^3^
ΔD	Hanna	29.9	0.73 × 10^7^	29.0	0.60 × 10^6^	0.7	1.58 × 10^6^
	Ingot	26.6	0.56 × 10^7^	25.4	1.38 × 10^6^	1.2	1.33 × 10^6^
	Otis	29.9	0.46 × 10^7^	26.4	2.09 × 10^6^	3.6	0.91 × 10^6^
	99S0155-14W	35.6	0.34 × 10^7^	31.2	2.78 × 10^6^	4.3	0.61 × 10^6^
	**LSD**	1.5	1.01 × 10^6^	2.0	4.80 × 10^5^	1.1	1.66 × 10^5^

HMW-AP: High Molecular Weight Amylopectin; LMW-AP: Low Molecular Weight Amylopectin; AM: Amylose; MWT: Molecular weight, measured as apparent average molecular weight by HPSEC; LSD: least significant difference (α = 0.05); ΔD: difference between non-sprouted and sprouted wheat.

## 4. Conclusions

Overall, PHS damage resulted in significant changes in physicochemical properties of the starch. Genotype and wheat seed coat color had significant effects on PHS score, α-amylase activity starch pasting and degradation of starch molecules. Mean α-amylase activities for HRS genotypes were lower than that for HWS genotypes, suggesting that in general, the HRS genotypes were less susceptible to PHS and starch degradation than the HWS genotypes. However, some HWS genotypes, including the AC Snowbird and 99S0155-14W exhibited a level of tolerance to PHS similar to HRS genotypes.
